# HOW FORMAL AND INFORMAL HEALTHCARE KNOWLEDGE IMPACT DEMENTIA CAREGIVER PREPAREDNESS

**DOI:** 10.1093/geroni/igae098.4210

**Published:** 2024-12-31

**Authors:** Zoe Thomas, Francesca Falzarano

**Affiliations:** University of Washington, Seattle, Washington, United States; University of Southern California, Los Angeles, California, United States

## Abstract

Dementia family caregivers frequently experience significant burden and depressive symptoms, which can harm their health and impair their ability to fulfill caregiving responsibilities. Previous research has shown that greater dementia knowledge or health literacy improves caregivers’ psychological symptoms and helps them feel more prepared for their role. Socioeconomic status and education are also strong predictors of health literacy. However, much of this research has been conducted in Asia, indicating there is a need to investigate dementia literacy further in the United States. This qualitative study aims to explore how formal versus informal healthcare system knowledge affects dementia family caregivers’ feelings of preparedness. Formal knowledge is defined as being obtained from a job or school education before becoming a caregiver. Informal knowledge includes doing independent research online, learning from support groups, and any past caregiving experience or managing personal healthcare. Knowledge as a theme emerged in 8 out of 30 caregiver semi-structured interviews, with excerpts subsequently analyzed and coded using reflexive thematic analysis. Results show that caregivers with formal knowledge of the healthcare system had a better grasp of dementia progression, felt more confident in discussions with physicians, and were better prepared to mitigate safety risks in the person with dementia. In contrast, those with informal knowledge displayed greater uncertainty and relied more heavily on the physician to validate their decisions. The findings highlight the advantages of formal healthcare knowledge in facilitating preparedness and indicate the need for educational interventions to improve health literacy among dementia family caregivers.

